# Reporting of the safety from allergic rhinitis trials registered on ClinicalTrials.gov and in publications: An observational study

**DOI:** 10.1186/s12874-022-01730-6

**Published:** 2022-10-05

**Authors:** Ivan Paladin, Shelly Melissa Pranić

**Affiliations:** 1 Department of ENT and Head and Neck Surgery, University Hospital of Split, Split, Croatia; 2 Department of Public Health, University of Split School of Medicine, Split, Croatia

**Keywords:** Allergic rhinitis, Randomised controlled trial, Adverse events, ClinicalTrials.gov, Completeness, Safety

## Abstract

**Background:**

Incomplete and inconsistent reporting of adverse events (AEs) through multiple sources can distort impressions of the overall safety of the medical interventions examined as well as the benefit-risk relationship. We aimed to assess completed allergic rhinitis (AR) trials registered in ClinicalTrials.gov for completeness and consistency of AEs reporting comparing ClinicalTrials.gov and corresponding publications.

**Methods:**

We retrospectively examined completed randomised controlled trials on AR registered in ClinicalTrials.gov on or after 9/27/2009 to trials updated with results on or before 12/31/2021 along with any corresponding publications. Complete reporting of AEs in ClinicalTrials.gov were summarised in tables describing AE information, and complete reporting in publications was an explicit statement of serious AE, death or other AE. Difference in completeness, number, or description of AEs between ClinicalTrials.gov and publication was classified as inconsistent reporting of AEs.

**Results:**

There were 99 registered trials with 45 (45.5%) available publications. All published trials completely reported AEs in ClinicalTrials.gov, and 21 (46.7%) in publications *(P < .001)*. In 43 (95.6%) publications, there was at least one inconsistency in the reporting of AEs *(P < .001)*. 8 (17.8%) publications had different number of serious AEs *(P = .003)*, 36 (80.0%) of other AEs *(P < .001)* while deaths reporting was inconsistent in 8 (57.1%) publications *(P = .127)*.

**Conclusion:**

The reporting of AEs from AR trials is complete in ClinicalTrials.gov and incomplete and inconsistent in corresponding publications. There is a need to improve the reporting of AEs from AR trials in corresponding publications, and thus to improve patient safety.

## Background

The reporting of adverse events (AEs) to ClinicalTrials.gov has been mandatory since September 2009 [1]. The Sect. 801 of the Food and Drug Administration Amendments Act (FDAAA 801) from 2007 and the Final Rule implemented in 2017 required investigators to report all anticipated and unanticipated Serious AEs (SAEs) and Other AEs (OAEs) data as well as All-Cause Mortality (ACM) data in tabular summaries [2]. These summaries reported in ClinicalTrials.gov should be identical to those reported in the corresponding publications but incomplete and inconsistent reporting of AEs in the publications is common, contrary to the Consolidated Standards of Reporting Trials (CONSORT) guidelines [3–7]. Such underreporting of AEs may result from the limitation of space imposed by journals, the use of study designs that measure harms poorly, or the deliberate concealment of unfavorable data [8–10]. Furthermore, it can minimise impressions about the overall safety of medical interventions and may distort the way decision-makers balance the benefit-risk relationship of those interventions [6, 11].

Global health and at the same time economic problems can be attributed to allergic rhinitis (AR), for which drugs are the mainstay therapy, require numerous clinical studies [12]. The data reporting from such studies should be complete and consistent across multiple sources to ensure the accuracy of evidence-based information that can be used by the lay or professional population [13–16]. Thus, we aimed to assess completed randomised controlled trials (RCTs) on AR registered in ClinicalTrials.gov for completeness of AEs reporting in ClinicalTrials.gov and for completeness and consistency in corresponding publications.

## Methods

### Study period and data sources

**Fig. 1:**
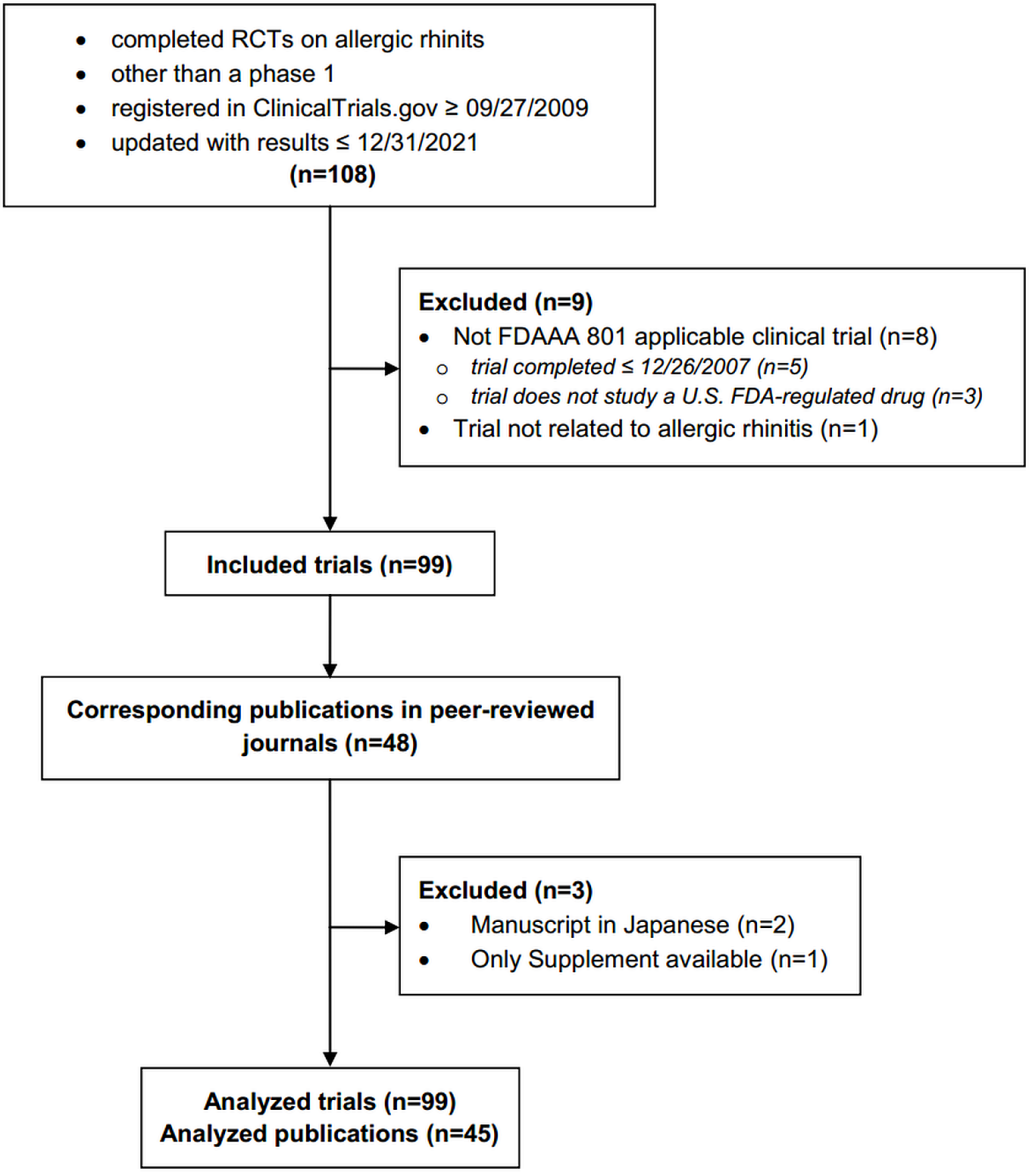
Study flowchart

We retrospectively analysed completed RCTs on AR shown in Fig. 1. The start date coincides with the date from which the reporting of adverse events became mandatory and the end date is recent and allowed more than 12 years of mandatory AEs reporting.

We searched ClinicalTrials.gov for completed RCTs using the following keywords: “allergic rhinitis”, “nasal allergies”, “rhinoconjunctivitis”, “hay fever”, and “atopic rhinitis”. We did not use Medical Subject Headings (MeSH) interventions. For trials with publications provided on ClinicalTrials.gov, we selected only those that reported the results of the current trial as the corresponding publications to the RCTs on AR. For trials without publications provided, we searched PubMed, Web of Science, Scopus and Google Scholar with the National Clinical Trial (NCT) identifier provided by ClinicalTrials.gov in the trial record that is usually listed in the abstract or main text of published articles [17]. If the initial search failed, we searched using the principal investigator’s name and study title. Only full publications were compared with registered data.

Flow diagram of retrospective cross-sectional study with inclusion and exclusion criteria. **FDAAA**: The Food and Drug Administration Amendment Act, **FDA**: The Food and Drug Administration.

### Sample

Inclusion criteria are shown in Fig. 1. Requirements for considering trials as applicable clinical trials according to the FDAAA 801 were verified according to “Elaboration of definitions of responsible party and applicable clinical trial (ACT)” from March 9, 2009 for trials initiated after September 27, 2007 and according to “Checklist for evaluating whether a clinical trial or study is an ACT” for those initiated after January 18, 2017 [18].

### Data extraction and comparisons

We primarily analysed the completeness and consistency of AEs reporting in ClinicalTrials.gov and the corresponding publications. The complete reporting of AEs in the registry were tables with the number of participants affected out of those at risk summarised for each AE, as required by the Final Rule [2]. Additionally, the “All-cause Mortality” item was not required before the Final Rule, therefore it was not analysed for trials with primary completion date before its implementation. We studied the reporting of the deaths of such trials from other elements of the outcome data, primarily from SAEs. In publications, the complete reporting of AEs was an explicit statement of the occurrence of SAEs, deaths, or OAEs according to the CONSORT extensions for better reporting harms in randomised trials [7]. Furthermore, any difference in the completeness, number of participants affected, number of AEs, or description of AEs between ClinicalTrials.gov and publication was classified as inconsistent reporting of AEs. Trial characteristics (phase, sponsor, specific dates, etc.) available at ClinicalTrials.gov as well as five-year journal impact factor were also analysed. Two investigators (IP and SP) independently extracted data in parallel from the entire cohort of trials and corresponding publications for the completeness and consistency of AEs reporting to avoid potential data collector bias from possible subjective interpretation. Inter-rater reliability was high for SAEs reporting between ClinicalTrials.gov and publications (kappa range 0.83 to 1.00). The inter-rater reliability was similarly high for OAEs reporting between the two sources (kappa range 0.79 to 0.91).We resolved through consensus discussion our interpretation of the differences in the number of SAEs reporting in publications which had the lowest kappa of 0.83 (95% confidence interval (CI) 0.65 to 1.01). Likewise, the inter-rater reliability for the OAEs reported as zero or non-occurring had the lowest kappa of 0.79 (95% CI 0.38 to 1.19) for which we resolved our interpretation through consensus discussion. We followed the Strengthening the Reporting of Observational Studies in Epidemiology (STROBE) guidelines for reporting observational studies [19]. There was no need for approval from an institutional review board (Ethics Committee of the University of Split School of Medicine), as this study is cross-sectional and historical cohort database study. We neither collected patient data nor performed experimental procedures.

### Statistical analysis

Data extracted from ClinicalTrials.gov were coded and then entered into a Microsoft Excel spreadsheet. Percentages, medians with the 95% CIs were presented. Two-level categorical variables (no or yes) were different SAEs description, SAEs reported in a publication, SAEs reported as zero in a publication, OAEs reported as Treatment-emergent AEs (TEAEs) and as Treatment-related AEs (TRAEs) in a publication. The number of OAEs, frequency threshold and elapsed time from posting results to publication were treated as nonparametric. We categorised these nonparametric values based on a median split, where values were dichotomised as 0, when less than or equal to their respective median, and as 1 when greater than their respective median. Differences between ClinicalTrials.gov and publications in the completeness and consistency of reporting of AEs, their reported number or description were compared using the Chi-square test or Mann-Whitney U test. Frequencies were compared using Chi-squared tests. We used IBM SPSS Statistics 21 (SPSS, Inc., an IBM company, Chicago, IL, USA, RRID:SCR_002865) for the analyses. Differences were considered significant at a *P* < .05.

## Results

### General characteristics

There were 99 registered trials with 45 (45.5%) available publications. Of the published trials, most were phase 3 (n = 21 [46.7%]), followed by phase 2 and 4 with 10 (22.2%) trials each. Furthermore, 34 (75.6%) of them were industry-sponsored trials, and 20 (44.4%)^1^ of these publications were published before the results of their trial were posted in ClinicalTrials.gov. The median five-year impact factor from the journals where these trials were published is 4.213 (95% CI 3.43–4.51). All 99 trials in ClinicalTrials.gov reported SAEs and OAEs, and all 17 trials whose primary completion date was after January 18, 2017 reported ACM data. In contrast, less than half of the publications (n = 21 [46.7%]) had a complete AEs report *(P < .001)*. In 43 (95.6%) publications, there was at least one inconsistency in the reporting of AEs compared to ClinicalTrials.gov *(P < .001)*. Table 1 shows discrepancies in reporting AEs between ClinicalTrials.gov and corresponding publications.

The withdrawal of participants due to AEs (median = 2, 95% CI 2.62–13.52) was explicitly stated in most publications (n = 43 [95.6%]). In 5 (11.1%) publications, the number of patients withdrawn due to AEs differed from the corresponding number in ClinicalTrials.gov, and in most cases (n = 4 [8.9%]) was higher than in ClinicalTrials.gov.

**Table 1:** Discrepancies in the reporting of AEs from AR trials between ClinicalTrials.gov and corresponding publications

	SAEs n (%)	OAEs n (%)	Deaths n (%)
	**Reporting rate**		
**ClinicalTrials.gov**	45 (100.0)	45 (100.0)	14 (100.0)^a^
**Publications**	34 (75.6)	44 (97.8)	6 (42.9)
***P*** **value** ^**b**^	*< 0.001*	*0.317*	*0.001*
	**AEs reported as zero**		
**ClinicalTrials.gov**	24 (53.3)	12 (26.7)	12 (85.7)
**Publications**	18 (40.0)	5 (11.1)	5 (35.7)
***P*** **value** ^**c**^	*0.001*	*< 0.001*	*0.014*
	**Number of reported AEs per trial (range)**		
**ClinicalTrials.gov**	0–28	0–10,648	0–1
**Publications**	0–15	0–2,769	0–1
***P*** **value** ^**b**^	*0.003*	*< 0.001*	*0.127*
	**Number of patients with AEs per trial (range)**		
**ClinicalTrials.gov**	0–23	0–1,031	0–1
**Publications**	0–11	0–1,215	0–1
***P*** **value** ^**b**^	*0.006*	*< 0.001*	*0.127*
	**Reported AEs per trial (median, 95% CI)**		
**ClinicalTrials.gov**	0 (95% CI 0.87–4.51)	21 (95% CI -60.86–1000.59)	0 (95% CI -0.07–0.35)
**Publications**	0 (95% CI 0.30–2.01)	77.5 (95% CI 57.78–338.31)	0 (95% CI -0.05–0.14)
	**Number of patients with AEs per trial (median, 95% CI)**		
**ClinicalTrials.gov**	0 (95% CI 0.78–3.88)	18 (95% CI 26.39–150.59)	0 (95% CI -0.07–0.35)
**Publications**	0 (95% CI 0.28–1.72)	59 (95% CI 54.75–188.73)	0 (95% CI -0.05–0.14)

*Abbreviations*: SAE – Serious Adverse Event, OAE – Other Adverse Event, CI – Confidence interval

^a^Sum of 6 pre-Rule trials and 9 post-Rule trials that had reported deaths

^b^Inconsistencies in reporting assessed by the Mann-Whitney Test with a significance set at *P < .05*

^c^Inconsistencies in reporting assessed by the Chi-square test with a significance set at *P* < .05

### SAEs reporting

In 8 (17.8%) publications, the number of reported SAEs differed from the corresponding number in ClinicalTrials.gov *(P = .003)*, of which 1 (2.2%) publication reported a higher number than in ClinicalTrials.gov, and 7 (15.6%) had a smaller number. The number of patients with at least one SAE differed in 7 (15.6%) publications compared to the corresponding number in ClinicalTrials.gov *(P = .006)*, and all 7 publications reported a smaller number than in ClinicalTrials.gov. In 15 (33.3%) publications the description of reported SAEs differed from the corresponding ones in ClinicalTrials.gov. Furthermore, trials in which less time elapsed from the posting of results in ClinicalTrials.gov to publication (less than or equal to the median of 3.4 months [95% CI -5.23–8.72]) were more prone to different descriptions of SAEs in publications, χ^2^ = 10.476, *P = .001*. Additionally, authors of publications corresponding to trials with zero reported SAEs in ClinicalTrials.gov were more likely to omit explicit reporting of SAEs in publications, χ^2^ = 4.746, *P = .029.*

### OAEs reporting

In 36 (80.0%) publications, the number of reported OAEs differed from the corresponding number in ClinicalTrials.gov *(P < .001)*, of which 12 (26.7%) publications reported a smaller number than in ClinicalTrials.gov, and 24 (53.3%) had a higher number. In 37 (82.2%) publications, the description of reported OAEs differed from the corresponding ones in ClinicalTrials.gov.17 (37.8%) publications reported OAEs only as TEAEs^2^ and 4 (8.9%) as TRAEs^3^. All publications that reported OAEs only as TEAEs had more reported OAEs than in ClinicalTrials.gov, and similarly all publications that reported OAEs only as TRAEs had less reported OAEs than in ClinicalTrials.gov, χ^2^ = 14.400, *P < .001* and χ^2^ = 9.000, *P = .003*, respectively. The number of patients with at least one OAE differed in 33 (73.3%) publications compared to the corresponding number in ClinicalTrials.gov *(P < .001)*. Of these, a smaller number of patients with OAEs were reported in 7 (15.6%) publications, and a higher number in 26 (57.8%). The median reported frequency threshold in ClinicalTrials.gov was 5% (95% CI 2.77–4.09) and in publications 0% (95% CI 0.58–2.11) indicating a lower frequency threshold report in publications than in ClinicalTrials.gov. Furthermore, 26 (57.8%) publications had different frequency thresholds, mostly (n = 24 [53.3%]) lower than in ClinicalTrials.gov. Additionally, significantly more publications with a lower frequency threshold than registered had more reported OAEs than in ClinicalTrials.gov, χ^2^ = 6.259, *P = .012*.

### Deaths reporting

35 (77.8%) published trials were completed before the implementation of the Final Rule, and 10 (22.2%) after. 17 (37.8%) pre-Rule trials reported deaths in corresponding publications while only 2 (4.4%) trials reported deaths as SAE and 2 (4.4%) through the ACM table in ClinicalTrials.gov. In contrast, all post-Rule trials reported deaths in ClinicalTrials.gov through the ACM table while only 4 (40.0%) corresponding publications reported deaths. Overall, 14 (31.1%) published trials reported deaths in ClinicalTrials.gov and 21 (46.7%) in publications. Thus, discrepancies in reporting of deaths was analysed for 14 (31.1%) published trials where we found that all of them reported deaths in ClinicalTrials.gov, and only 6 (42.9%) in corresponding publications as well *(P = .001)* (Table 1). The published number of the reported deaths differed from the registered number in only 1 (2.2%)^4^ pre-Rule trial where the reporting of deaths in the publication was omitted due to reporting of AEs only as TRAEs *(P = .127)*.

## Discussion

Our novel cross-sectional study on the discrepancies in the reporting of safety data from RCTs on AR showed incomplete and inconsistent AEs reporting in corresponding publications. Most published trials were phase 2, 3 and 4 (n = 42 [93.3%]) and were published in high-impact journals. Therefore such publications should contain consistent and complete data in relation to the registry as they have the greatest impact on clinical care and formulation of clinical practice guidelines [20]. A similar problem of safety underreporting that raises concerns about using journal publications is well-documented, and limited space in journals has been cited as one of the more common reasons [6, 8, 21]. The question is whether such a reason for omitting reporting of SAEs and deaths is justified because the statement “there were no SAEs or deaths” does not take up much space. Authors who have previously studied discrepancies in the reporting of AEs and other results have concluded that there is a need to change and implement regulatory requirements for timely and complete posting of results, including clearer AEs reporting [22]. Furthermore, an additional checklist was suggested during submission to the journal where authors should explain any possible discrepancies with registered data and provide a link to the appropriate ClinicalTrials.gov record to help journal editors find discrepancies between registered data and data in submitted manuscripts [20]. Additionally, the registry interface could be updated so that trial registration cannot be completed until all required fields are filled.

### SAEs reporting

Almost a quarter of the analysed publications did not explicitly report SAEs, and it is evident that publications mostly underreported SAEs as well as participants affected by SAEs in relation to ClinicalTrials.gov. As already stated in the results, one of the reasons for omitting explicit reporting of SAEs is definitely their absence during the trial, reported as zero in ClinicalTrials.gov. A number of other authors have found similar discrepancies in SAEs reporting between ClinicalTrials.gov and corresponding publications [3–6]. The fact that 20 trials were published prior to posting results in ClinicalTrials.gov is probably the reason for the statistical significance in the difference between the elapsed time from posting results to publication date and the different description of SAEs in the publication.

### OAE reporting

Only one (2.2%) publication omitted the reporting of OAEs but most (n = 37 [82.2%]) had inconsistent reporting compared to ClinicalTrials.gov. Unlike SAEs underreporting, we found the overreporting of OAEs as well as participants affected by OAEs in the publications. One of the reasons for reporting more OAEs in publications is a lower frequency threshold at which to report OAEs in more than half of the publications. A similar conclusion was reached in the study by *Jurić et al.* [22]. Discrepancies in the reporting of OAEs and overreporting of affected participants in publications were also described by *Hartung et al.*. who, unlike the present study, found underreporting of OAEs in their analysed publications [6]. We have found that the underreporting of OAEs in publications were present if OAEs were reported as TRAEs. Most authors have studied discrepancies in the reporting of SAEs [3–6], but very few have studied the consistency of reporting OAEs which also represent the safety of investigational drugs and clinical studies. Consistent reporting of OAEs across multiple sources also contributes to patient safety, therefore further studies are needed.

### Deaths reporting

The analysis of the reporting of deaths differed from the analysis of remaining adverse events due to different regulations and requirements in their reporting during the analysed period. Therefore, discrepancies in deaths reporting has been analysed in less than a third of published trials, and the underreporting of deaths found in publications supports the results of other studies that analysed discrepancies in the reporting of AEs between trial registry and publications [6, 23]. The results also show that 15 (33.3%) more publications reported deaths while that report was missing in ClinicalTrials.gov. The reporting of deaths is an ethical responsibility to uphold the transparent reporting of patient data, and regardless of whether the time frame of the reporting of deaths was before or after the Final Rule, the reporting of deaths from this study was similar to the rate from other studies from nearly 10 years ago [6, 23]. However, further and larger studies are needed.

### Study limitations

We analysed trials only registered in ClinicalTrials.gov. There are currently 17 other primary registries in the World Health Organization (WHO) registry network that meet International Committee of Medical Journal Editors (ICMJE) requirements and therefore our analysed data may be incomplete and inaccurate [24]. However, we used ClinicalTrials.gov, which is the largest clinical trial registry [25, 26]. Another limitation could be the oversight of some of the existing publications despite different search methods. Finally, our samples regarding RCTs and publications were small so the discrepancies recorded should be viewed with caution.

## Conclusion

The reporting of AEs from completed and published AR trials is complete in ClinicalTrials.gov and incomplete and inconsistent in corresponding publications despite all recommendations and guidelines. The SAEs and deaths were underreported in publications while OAEs were overreported. Inconsistent and incomplete reporting of AEs in publications compromises patient safety so there is a need for a more detailed comparison of registered and submitted AEs during the publication process.

## Data Availability

The datasets generated and/or analysed during the current study are available in the Open Science Framework repository, 10.17605/OSF.IO/QVP62. Competing interests (Declaration of Conflicting Interests). The authors declare that they have no competing interests.

## Footnotes

1 *NCT02696850, NCT01861522, NCT01783548, NCT01700192, NCT01697956, NCT01644617, NCT01586091, NCT01380327, NCT01307319, NCT01270256, NCT01185080, NCT01134705, NCT01010971, NCT01007253, NCT01003301, NCT01424397, NCT03705793, NCT03682965, NCT01660698, NCT03394508*.

2 *NCT02870205, NCT02709538, NCT02631551, NCT02318303, NCT01817790, NCT01794741, NCT01697956, NCT01413958, NCT01401465, NCT01330017, NCT01307319, NCT01154153, NCT01134705, NCT01133626, NCT01024608, NCT01010971, NCT00988247*.

3 *NCT01783548, NCT01644617, NCT01385371, NCT01231464*.

4 *NCT01385371*.

## Declarations

Furthermore, all data used in this study are publicly available in the ClinicalTrials.gov registry and therefore no permission was required to access the data.

This study neither studied nor analysed the raw data of other studies. We analysed their data which were publicly registered in ClinicalTrials.gov and published in publicly available scientific journals. So no permission was needed here either.

The patient data from the registered studies in the ClinicalTrials.gov registry, which we analysed, were summarised and thus anonymised by their authors, so no anonymisation was required in this study.

## Abbreviations

AE: Adverse Event.
AR: Allergic rhinitis.
CONSORT: Consolidated Standards of Reporting Trials.
FDAAA: The Food and Drug Administration Amendment Act.
ICMJE: International Committee of Medical Journal Editors.
MeSH: Medical Subject Headings.
NCT: National Clinical Trial.
OAE: Other Adverse Event.
RCT: Randomised controlled trials.
SAE: Serious Adverse Event.
STROBE: Strengthening the Reporting of Observational Studies in Epidemiology.
TEAE: Treatment-emergent Adverse Event.
TRAE: Treatment-related Adverse Event.
TRDS: Trial Registration Data Set.
WHO: World Health Organization.
